# Supplementary material for: A single m6A modification in U6 snRNA diversifies exon sequence at the 5’ splice site
Source: Nat Commun. 2021 May 28;12:3244. doi: 10.1038/s41467-021-23457-6 (PMC8163875; doi:10.1038/s41467-021-23457-6)
Supplement: Supplementary file 1 — Supplementary Information [file 41467_2021_23457_MOESM1_ESM.pdf]

Supplementary information

A single m^{6}A modification in U6 snRNA diversifies exon sequence at 5' splice site

Yuma Ishigami ***et al.***

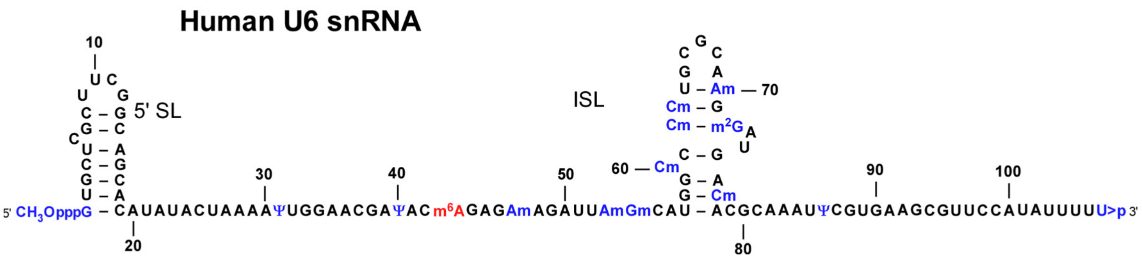

Supplementary Figure 1. **Post-transcriptional modifications of human U6 snRNA.**

Primary and secondary structures of human U6 snRNA with post-transcriptional modifications: CH3OpppG (γ-methyl triphosphate cap), N^6-methyladenosine (m^6A), pseudouridine (Ψ), 2'*O*-methylations (Nm), and 2',3' cyclic phosphate (>p). 5'SL and ISL represent the 5' stem loop and internal stem loop, respectively.

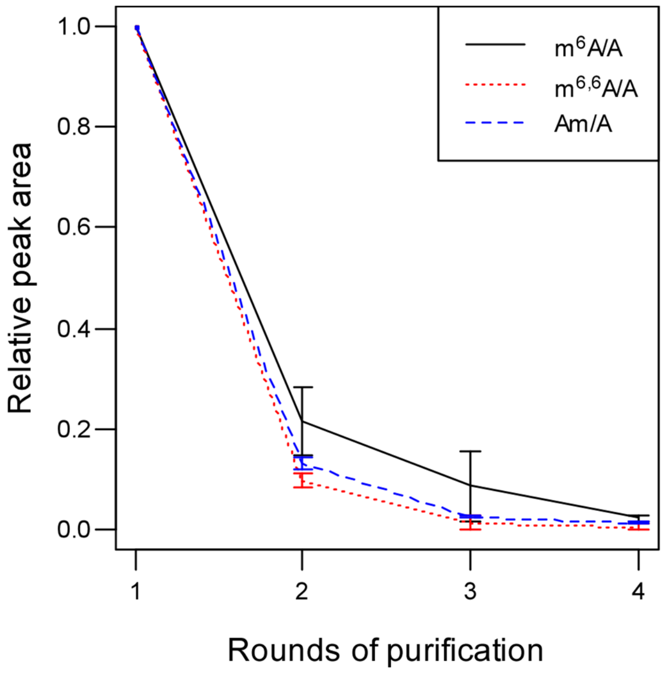

Supplementary Figure 2. Nucleoside analysis of *S. pombe* mRNAs at each step of poly(A)$^{+}$ RNA purification

The abundance of m^{6}A and rRNA modification (m^{6,6}A and Am) are monitored by LC/MS/MS in the course of poly(A)$^{+}$ RNA purification. The mass chromatogram area ratio of the modified adenosines against unmodified adenosine were calculated as relative peak area at each step. The differences between peak area of m^{6}A and the other modifications were not significant from 2nd to 4th rounds of purifications. Data are presented as mean values +/- SD. n=3 biologically independent samples. Source data are provided as a Source Data file.

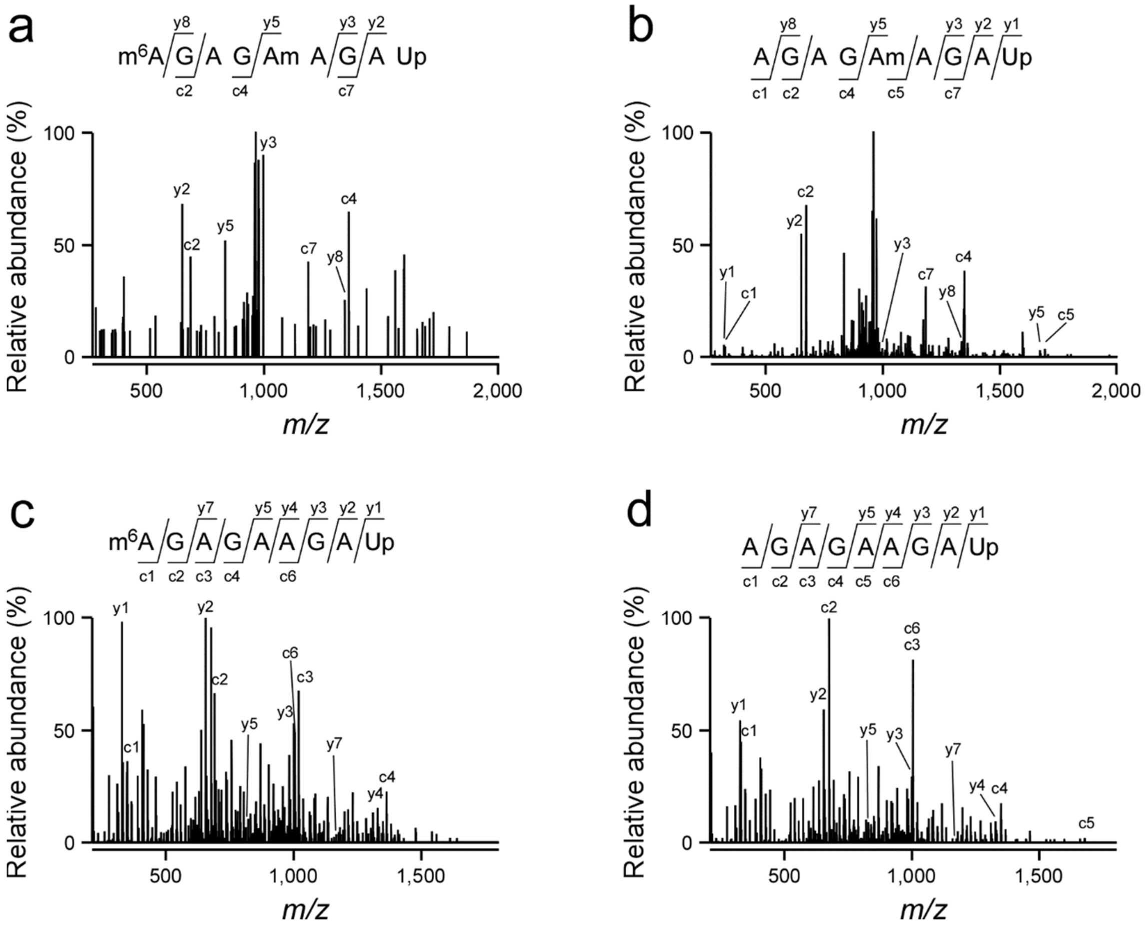

Supplementary Figure 3. **CID spectra of RNA fragments from *S. pombe* U6 snRNA.** (a and b) CID spectra of the RNase A-digested fragments of U6 snRNAs isolated from *S. pombe* WT (a) and *mtl16*Δ (b) strains. (c and d) The same fragments of the U6 snRNA transcript, with (c) or without (d) m^6A, reconstituted *in vitro*. The triply-charged negative ions of the RNA fragments [*m/z* 1010.15 (a), 1005.48 (b,c), 1000.81 (d)] were used as the precursors for CID. Product ions are assigned on the sequence of each fragment.

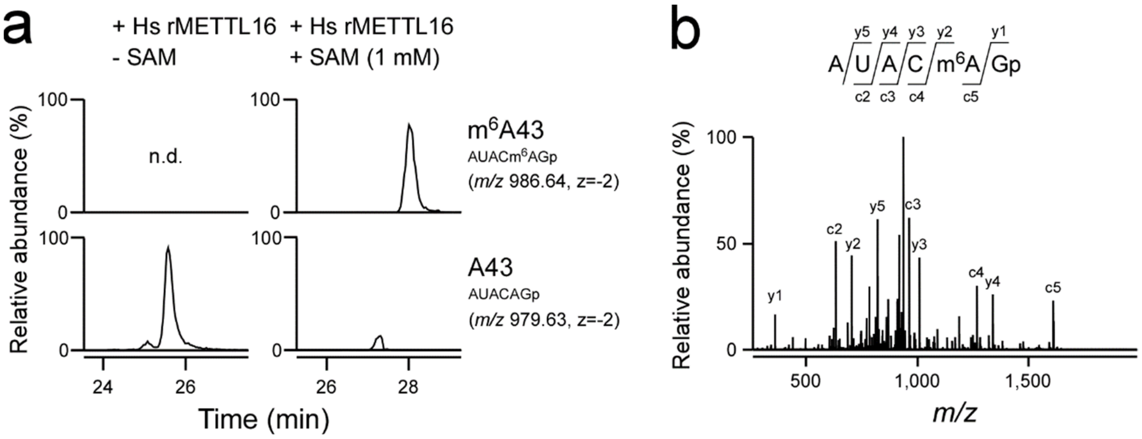

Supplementary Figure 4. *In vitro* reconstitution of m^{6}A in U6 snRNA catalyzed by human METTL16.

(a) *In vitro* reconstitution of m^{6}A in U6 snRNA catalyzed by human recombinant METTL16 in the presence or absence of SAM. Mass chromatograms show the RNase T_1–digested fragments of U6 snRNA transcripts containing m^{6}A43 (upper panels) or A43 (lower panels).

(b) CID spectra of the RNase T_1-digested fragments of U6 snRNA transcript with m^{6}A reconstituted *in vitro*. The doubly-charged negative ion of the RNA fragments (*m/z* 986.64) was used as the precursor for CID. Product ions are assigned on the sequence of the fragment.

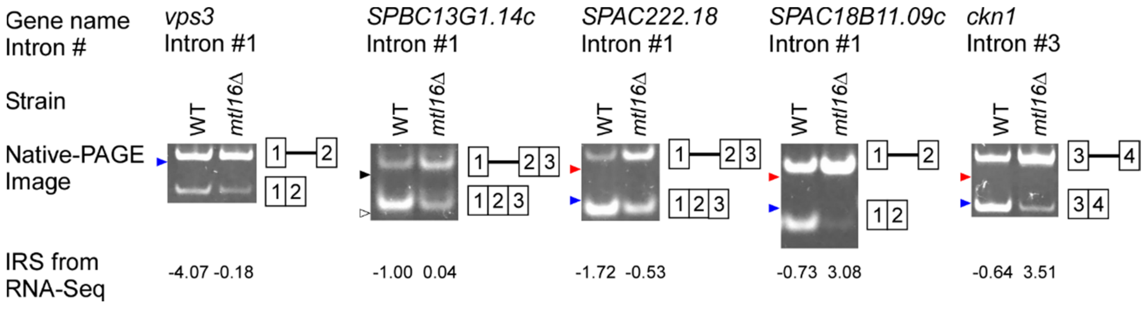

Supplementary Figure 5. Semi-quantitative RT-PCR of introns with large IRS differences in the WT and *mtl16*Δ strains.

Upper and lower gel bands represent retained and spliced introns, respectively. IRS values (calculated from RNA-seq) are written below the gel images. Blue, red, white, and black arrows represent the positions of DNA ladders with lengths of 150, 200, 300, and 350 bp, respectively. Source data are provided as a Source Data file.

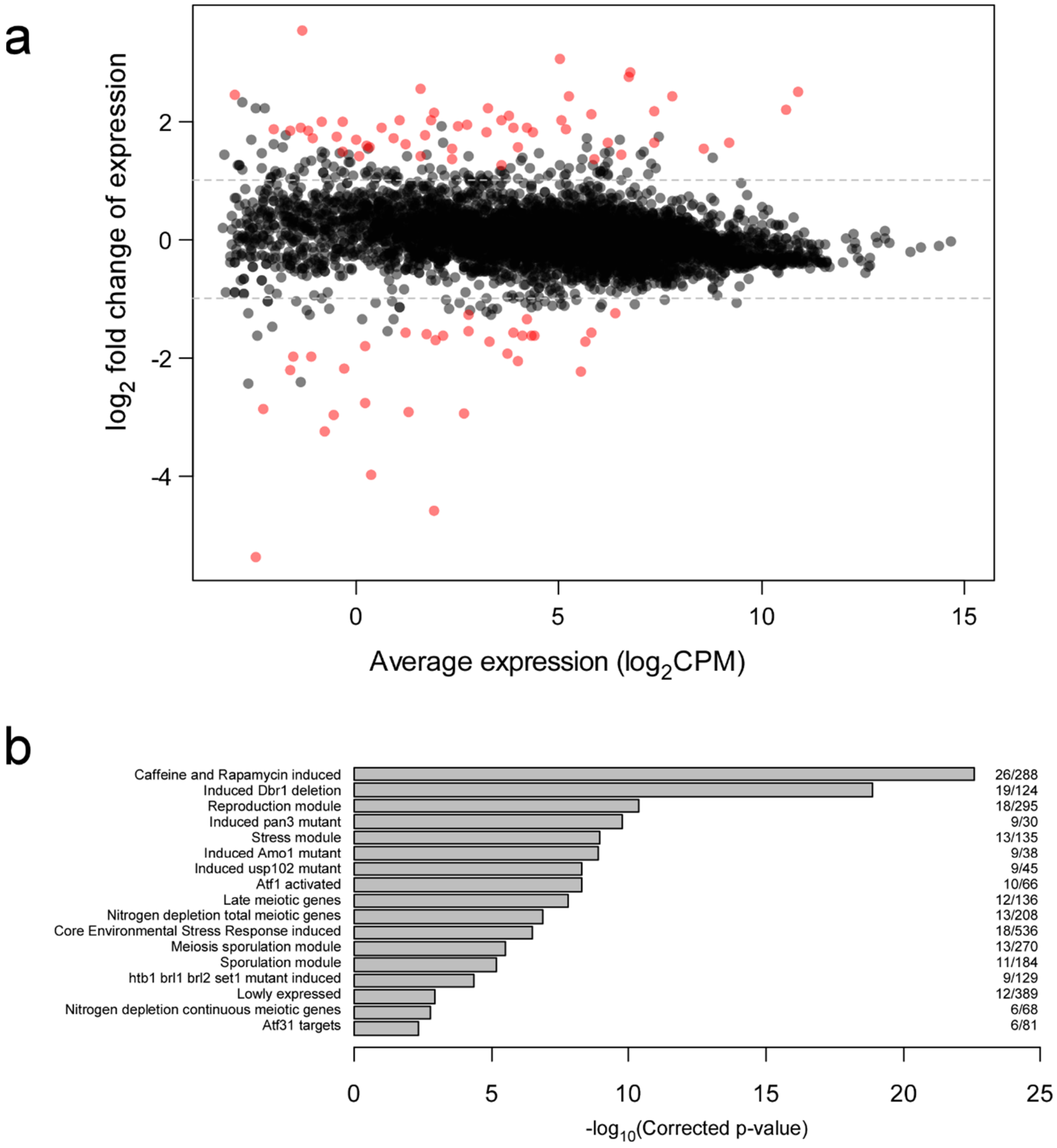

Supplementary Figure 6. Differential expression analysis upon *mtl16* knockout.

(a) MA plot of differential expression between the WT and *mtl16*Δ strains. Red plots indicate genes significantly up- or down-regulated over 2-fold.

(b) Gene ontology analysis of the genes that were up-regulated in the *mtl16*Δ strain.

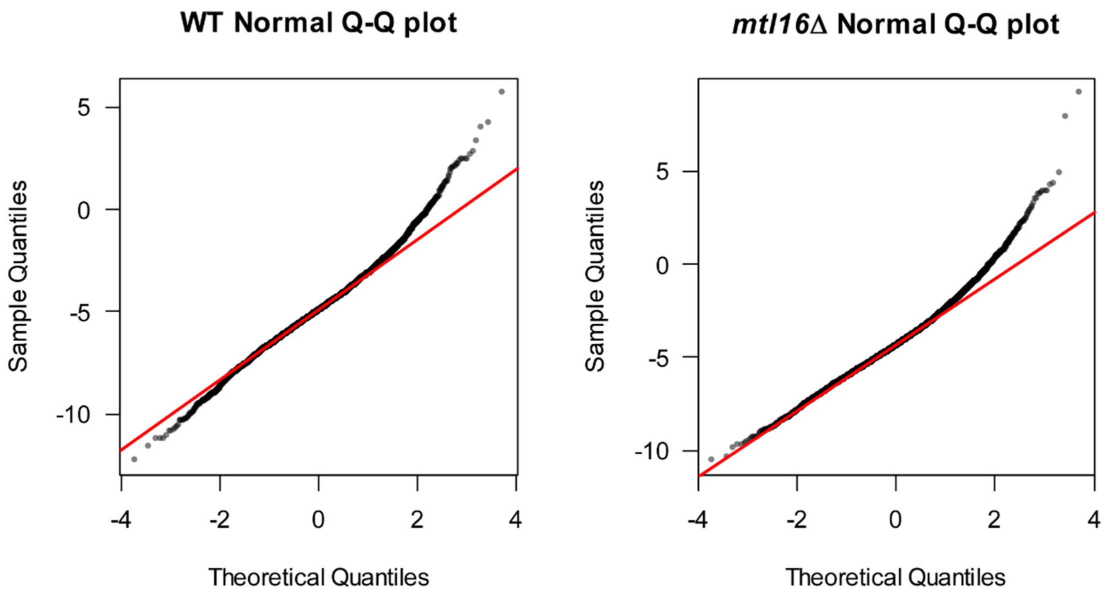

Supplementary Figure 7. IRS distribution evaluated by Quantile-Quantile plot. Q-Q plot of IRS values from each quadruplicate of the WT or *mtl16*Δ strain. The IRS values were plotted against their quantiles as black dots. The red line represents the theoretical normal distribution which connects the first and third quartiles.

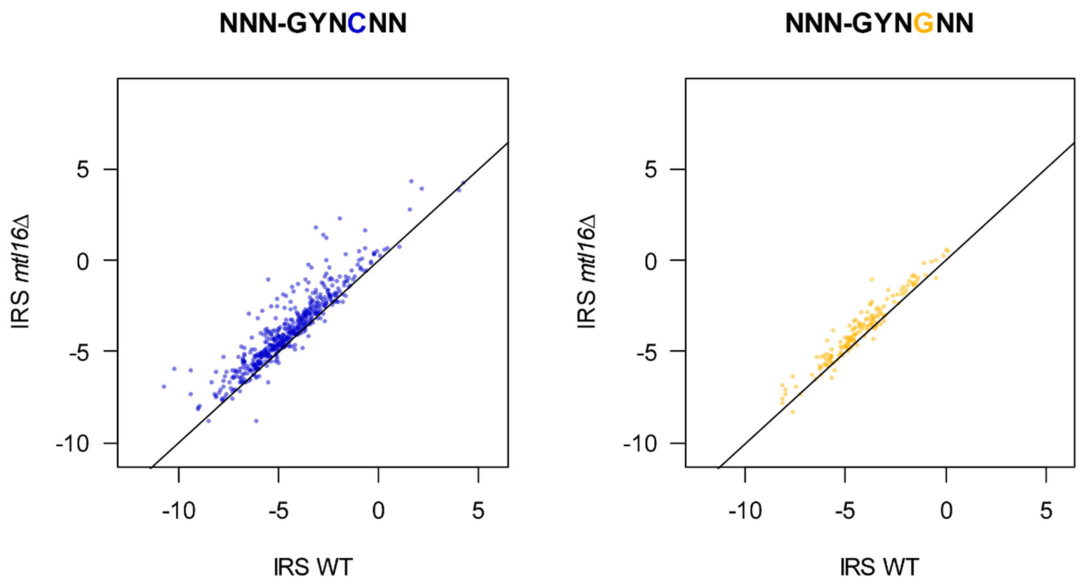

Supplementary Figure 8. Scatter plots of IRS of C4 and G4 introns.

Scatter plots of IRS of C4 and G4 introns in *mtl16*Δ *versus* WT. The black lines represent an equal value of IRS.

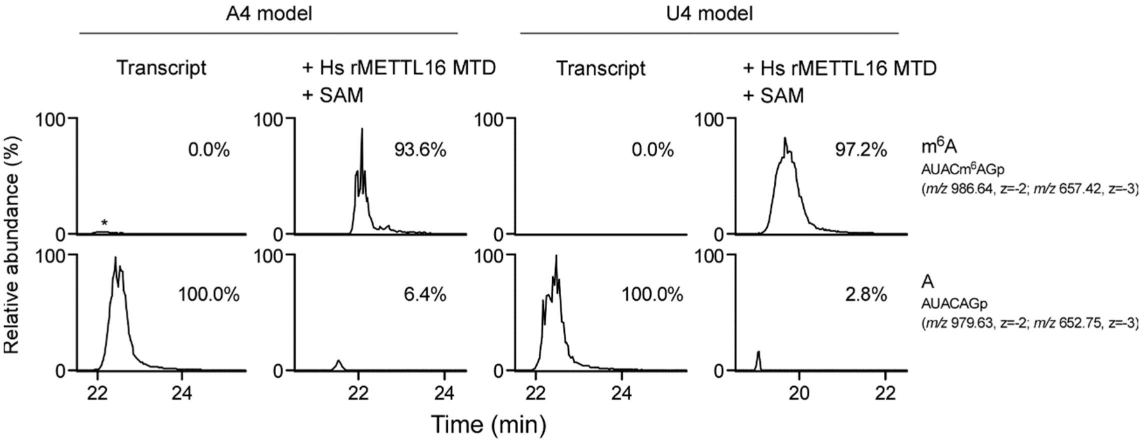

Supplementary Figure 9. ***In vitro*** **methylation of the model RNA substrates.**

In vitro methylation of model RNA hairpins catalyzed by recombinant METTL16 methyltransferase domain (MTD). RNase T_1-digested fragments of methylated or unmodified RNAs are analyzed by LC/MS. Mass chromatograms of the doubly- and triply-charged ions of each fragment are described. Percentages represent the relative peak area of each fragment. The asterisk represents a nonspecific peak.

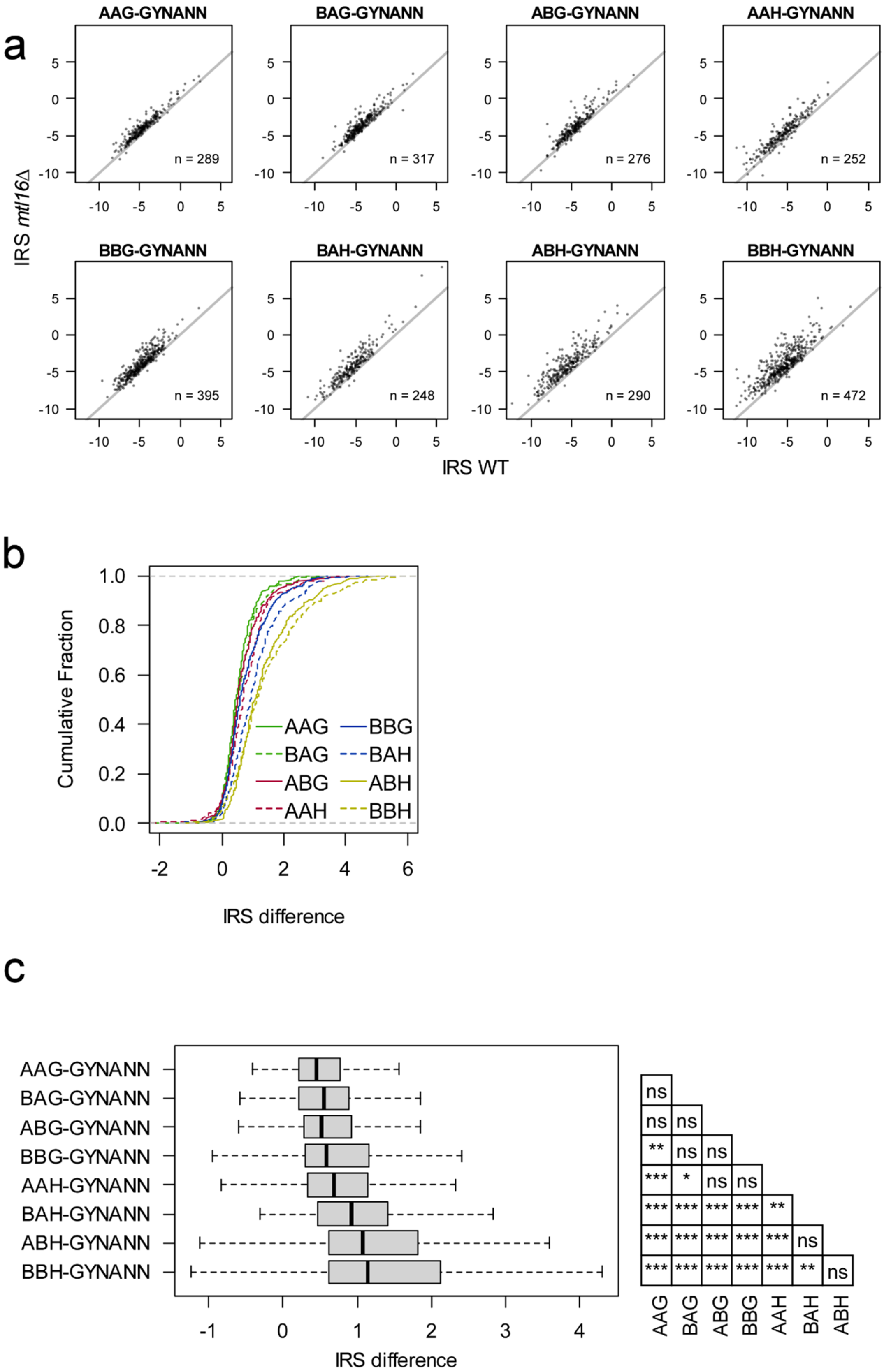

ns						
ns	ns					
**	ns	ns				
***	*	ns	ns			
***	***	***	***	**		
***	***	***	***	***	ns	
***	***	***	***	***	**	ns

Supplementary Figure 10. Plots for A4 introns with different 5’ exon sequences

(a) IRS plots for A4 introns with different 5’ exon sequences in *mtl16Δ versus* WT. The gray lines represent an equal value of IRS.

(b) Cumulative plot of IRS differences for each group of classified introns in *mtl16Δ versus* WT. All introns are classified into eight groups based on their 5’ exon triplet sequence.

(c) Box plots showing the IRS difference for each group of A4 introns in *mtl16Δ versus* WT. The inside box shows the interquartile ranges, the black vertical thick line represents the median, and the whiskers represent the 1.5 × interquartile ranges. The p-value of different distributions (two-sided Wilcoxon’s rank–sum test adjusted by Bonferroni correction) is shown on the right. *p = 0.03, **p < 0.01, ***p < 0.0005. Number of samples are shown in (a).

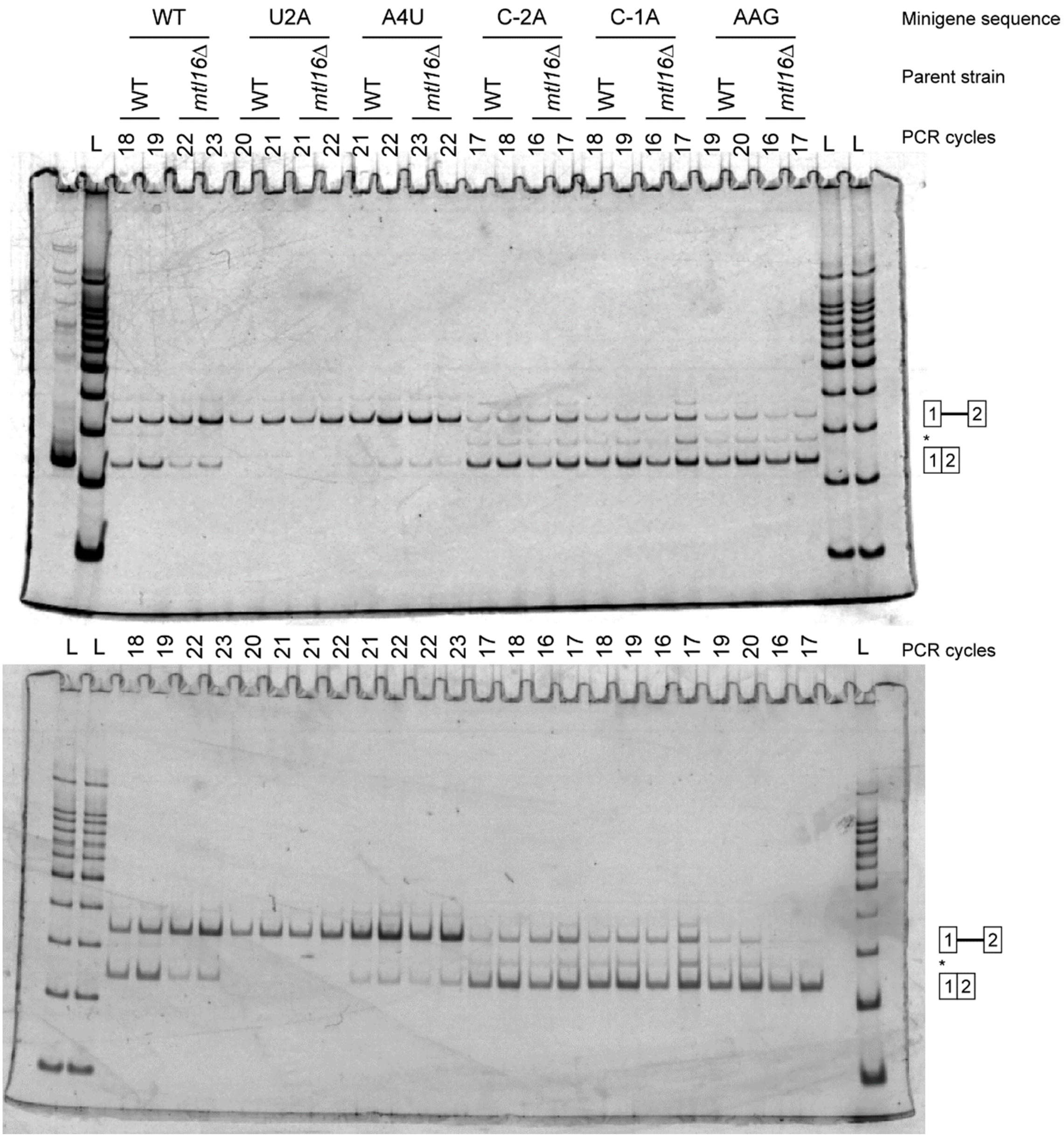

Supplementary Figure 11. Gel image of minigene semi-quantitative RT-PCR.
The corresponding isoforms of the bands are shown on the right side. Non-specific bands are marked by an asterisk. Bands derived from each isoform was quantified to calculate IRS for each minigene sequence in each strain, using ladders as a standard. The upper and lower gels are technical replicates. Lanes marked with L are loaded with a Toyobo 100 bp DNA ladder (sizes from bottom are 100, 200, 300, 400, 500, 600, 700, 800, 900, 1000, and 1500 bp).

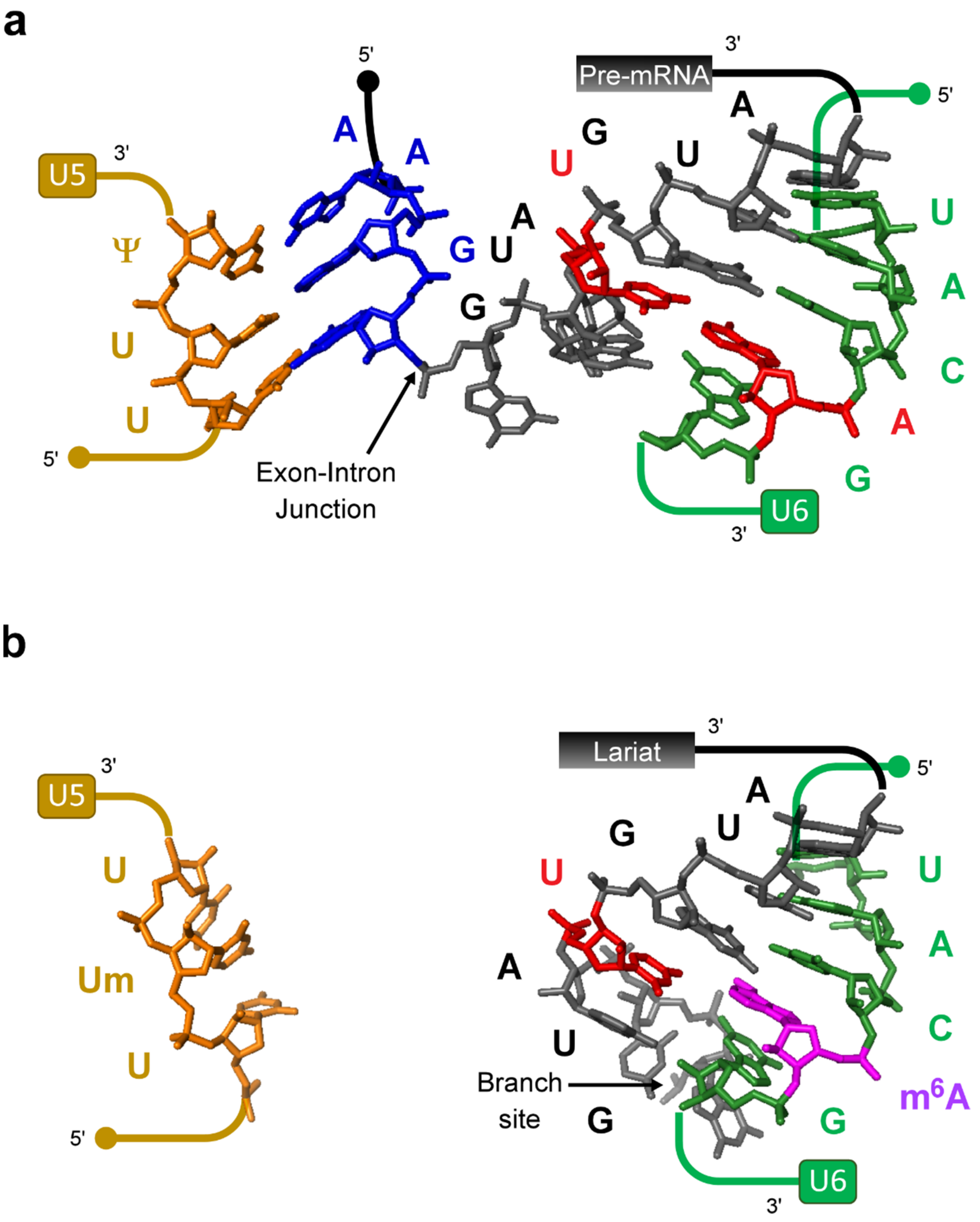

Supplementary Figure 12. CryoEM structures of 5'SS.

(a) *S. cerevisiae* pre-catalytic spliceosome (B^{act} complex, PDB ID:5GM6 [http://doi.org/10.2210/pdb5gm6/pdb]) [1]

(b) *S. pombe* ILS complex (PDB ID:3JB9 [http://doi.org/10.2210/pdb3jb9/pdb]) [2].

The model consists of U5 snRNA (orange), U6 snRNA (green), 5' exon (blue), intron (black), A4 (red), and m^6A (magenta). The coordinates of the methyl group of m^6A are set to the *syn* conformation.

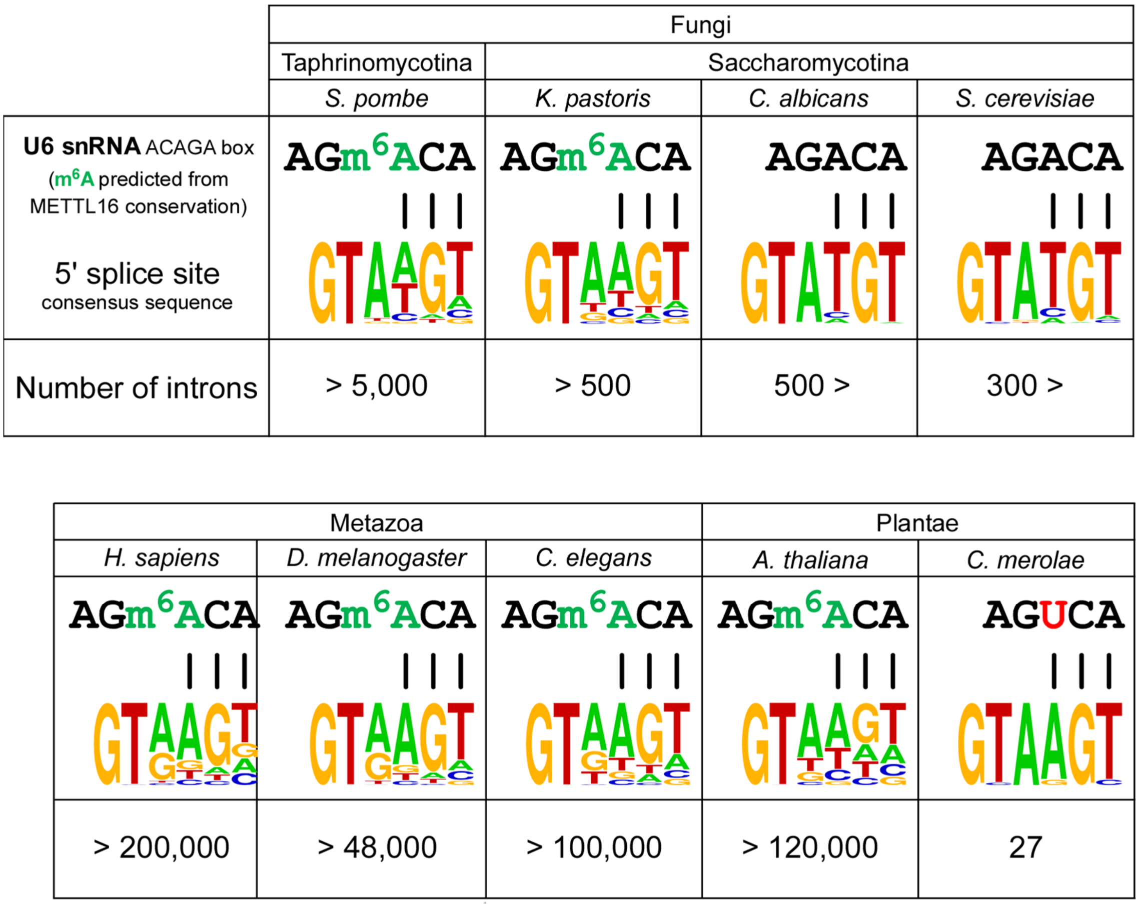

Supplementary Figure 13. Evolutionary comparison of the 5'SS consensus sequence and the AGACA box of U6 snRNA.

In each organism, the ACAGA box including m^{6}A modification (green) base pairs with the 5'SS consensus sequence depicted by Logos. The number of introns is described in each organism.

Supplemental References

1 Yan, C., Wan, R., Bai, R., Huang, G. & Shi, Y. Structure of a yeast activated spliceosome at 3.5 A resolution. *Science* **353**, 904-911, doi:10.1126/science.aag0291 (2016).

2 Yan, C. *et al.* Structure of a yeast spliceosome at 3.6-angstrom resolution. *Science* **349**, 1182-1191, doi:10.1126/science.aac7629 (2015).
