# Supplementary material for: A single m6A modification in U6 snRNA diversifies exon sequence at the 5’ splice site
Source: Nat Commun. 2021 May 28;12:3244. doi: 10.1038/s41467-021-23457-6 (PMC8163875; doi:10.1038/s41467-021-23457-6)
Supplement: Supplementary file 8 — Description of Additional Supplementary Files [file 41467_2021_23457_MOESM8_ESM.docx]

Description of additional supplementary information

Title: Supplementary_Data_1

Description: Splicing analysis results and raw data Explanation for each column are as follows:

ID Unique number CHR Chromosome Name INTRST Start coordinate of Intron INTREN End coordinate of Intron STRAND Strand of Intron GENEID Gene ID of which the Intron belongs GENENAME Gene Name of which the Intron belongs TRANID Transcript ID of which the Intron belongs INTRNUM Intron index in the transcript s5p Nucleotide sequence of intron 5' end s3p Nucleotide sequence of intron 3' end minreads minimal cov(Total) among WT1-4 and KO1-4 conirsme Mean IRS in WT strain treirsme Mean IRS in KO strain conirssd Unbiased standard deviation of WT IRS treirssd Unbiased standard deviation of KO (mtl16Δ) IRS irsdiff Difference in mean IRS between WT and KO irszscor e Z-score of IRS n5pe3 Genomic nucleotide at position -3 from the 5' splice site n5pe2 Genomic nucleotide at position -2 from the 5' splice site n5pe1 Genomic nucleotide at position -1 from the 5' splice site n5pi4 Genomic nucleotide at position 4 from the 5' splice site seq20i Genomic sequence of the intron with 20 basepairs from the flanking exons seq5p15 15 bp sequence around the 5' splice site seq3p23 23 bp sequence around the 3' splice site conpcsme Mean PCS in WT strain trepcsme Mean PCS in KO strain eijrCon1 -4 Read number from WT quadruplicates 1-4 classified as EIJR eijrTre1 -4 Read number from KO quadruplicates 1-4 classified as EIJR iejrCon1 -4 Read number from WT quadruplicates 1-4 classified as IEJR iejrTre1 -4 Read number from KO quadruplicates 1-4 classified as IEJR csrCon1- 4 Read number from WT quadruplicates 1-4 classified as CSR csrTre1- 4 Read number from KO quadruplicates 1-4 classified as CSR a5rCon1- 4 Read number from WT quadruplicates 1-4 classified as A5R a5rTre1- 4 Read number from KO quadruplicates 1-4 classified as A5R a3rCon1- 4 Read number from WT quadruplicates 1-4 classified as A3R a3rTre1- 4 Read number from KO quadruplicates 1-4 classified as A3R irs_con1 -4 IRS from WT quadruplicates 1-4 irs_tre1 -4 IRS from KO quadruplicates 1-4

Title: Supplementary_Data_2

Description: Differential gene expression upon deletion of mtl16 (a) Steady-state levels of protein-coding genes significantly increased over 2-fold in the mtl16Δ strain. logFC=log2 fold-change of expression, logCPM=log2 counts per million, PValue=unadjusted p-value of two-sided quasi-likelihood F test, FDR=False Discovery Rate. (b) Steady-state levels of protein-coding genes significantly decreased over 2-fold in the mtl16D strain. (c) Differential expression analysis result of all annotated genes

Title: Supplementary_Data_3

Description: List of primers, probes, strains, and vectors.

Title: Supplementary_Data_4

Description: Trimming and mapping statistics

Title: Supplementary_Data_5

Description: AnGeLi Results of upregulated genes Corrected_pvalue is the p-value of two-sided Fisher's exact test corrected for multiple testing by the FDR method.
