# Supplementary material for: A single m6A modification in U6 snRNA diversifies exon sequence at the 5’ splice site
Source: Nat Commun. 2021 May 28;12:3244. doi: 10.1038/s41467-021-23457-6 (PMC8163875; doi:10.1038/s41467-021-23457-6)
Supplement: Supplementary file 9 — Reporting Summary [file 41467_2021_23457_MOESM9_ESM.pdf]

nature research

Corresponding author(s): Tsutomu Suzuki

Last updated by author(s): Apr 21, 2021

Reporting Summary

Nature Research wishes to improve the reproducibility of the work that we publish. This form provides structure for consistency and transparency in reporting. For further information on Nature Research policies, see our Editorial Policies and the Editorial Policy Checklist.

Statistics

For all statistical analyses, confirm that the following items are present in the figure legend, table legend, main text, or Methods section.

n/a	Confirmed	
☐	☒	The exact sample size (*n*) for each experimental group/condition, given as a discrete number and unit of measurement
☐	☒	A statement on whether measurements were taken from distinct samples or whether the same sample was measured repeatedly
☐	☒	The statistical test(s) used AND whether they are one- or two-sided<br>*Only common tests should be described solely by name; describe more complex techniques in the Methods section.*
☒	☐	A description of all covariates tested
☐	☒	A description of any assumptions or corrections, such as tests of normality and adjustment for multiple comparisons
☐	☒	A full description of the statistical parameters including central tendency (e.g. means) or other basic estimates (e.g. regression coefficient) AND variation (e.g. standard deviation) or associated estimates of uncertainty (e.g. confidence intervals)
☐	☒	For null hypothesis testing, the test statistic (e.g. *F*, *t*, *r*) with confidence intervals, effect sizes, degrees of freedom and *P* value noted<br>*Give P values as exact values whenever suitable.*
☒	☐	For Bayesian analysis, information on the choice of priors and Markov chain Monte Carlo settings
☒	☐	For hierarchical and complex designs, identification of the appropriate level for tests and full reporting of outcomes
☒	☐	Estimates of effect sizes (e.g. Cohen's *d*, Pearson's *r*), indicating how they were calculated

Our web collection on statistics for biologists contains articles on many of the points above.

Software and code

Policy information about availability of computer code

Data collection	RNA-seq raw data was obtained by NovaSeq (150-bp, paired-end).<br>Yeast colony images were obtained by FAS-III.<br>Gel images were obtained by FLA-7000.<br>RT-qPCR data was obtained by LightCycler480.<br>Public databases: Ensembl, PomBase.
Data analysis	Canvas 15, Excel 2016 and R 4.0.3 were used to draw figures and analyze statistical data.<br>Xcalibur 4.1 was used for mass spec analysis.<br>Multi Gauge V3.0 was used for graphical analysis.<br>fastp 0.20.0 was used for raw read quality control and adapter trimming.<br>Trimmed reads were mapped to S. pombe genome assembly ASM294v2.34 using STAR 2.7.3a.<br>Junction reads were analyzed by a custom python script [https://github.com/YumaIshigami/irscalc] running on Python 3.8.5 with libraries Pysam 0.15.2 and Biopython 1.72.<br>Gene expression data was analyzed by HTSeq 0.6.1 and EdgeR 3.12.<br>Pymol 2.1.0 was used to draw structure data.

For manuscripts utilizing custom algorithms or software that are central to the research but not yet described in published literature, software must be made available to editors and reviewers. We strongly encourage code deposition in a community repository (e.g. GitHub). See the Nature Research guidelines for submitting code & software for further information.

Data

Policy information about availability of data

All manuscripts must include a data availability statement. This statement should provide the following information, where applicable:

- Accession codes, unique identifiers, or web links for publicly available datasets
- A list of figures that have associated raw data
- A description of any restrictions on data availability

The sequence data from this study have been submitted to the DDBJ Sequence Read Archive under accession number DRA009909 [https://ddbj.nig.ac.jp/DRASearch/submission?acc=DRA009909]. Structural data was retrieved from PDB ID: 5O9Z [http://doi.org/10.2210/pdb5o9z/pdb], 5GM6 [http://doi.org/10.2210/pdb5gm6/pdb] and 3JB9 [http://doi.org/10.2210/pdb3jb9/pdb].

Field-specific reporting

Please select the one below that is the best fit for your research. If you are not sure, read the appropriate sections before making your selection.

☒ Life sciences ☐ Behavioural & social sciences ☐ Ecological, evolutionary & environmental sciences

For a reference copy of the document with all sections, see nature.com/documents/nr-reporting-summary-flat.pdf

Life sciences study design

All studies must disclose on these points even when the disclosure is negative.

Sample size	No statistical methods were used to predetermine sample size. Four biologically independent samples for each strain were applied for sequencing analyses, based on standards in the field. For Nucleoside analysis, RT-PCR, RT-qPCR, growth assay and melting temperature observation assay, sample size was three or above to apply Student's t-test.
Data exclusions	For splice junction analysis in RNA-seq, total coverage of a junction supported by under 10 reads in WT or KO conditions were excluded from further analyses. The exclusion criteria were pre-established.
Replication	Reproducibility for RNA-seq data and analyses were confirmed by four independent biological replicates. All attempts at other low-throughput experiments were successful.
Randomization	Randomization was not required in this study because there is no statistic that requires randomization of samples.
Blinding	Blinding was not required in this study because it would not increase the reliability of the experiments.

Reporting for specific materials, systems and methods

We require information from authors about some types of materials, experimental systems and methods used in many studies. Here, indicate whether each material, system or method listed is relevant to your study. If you are not sure if a list item applies to your research, read the appropriate section before selecting a response.

Materials & experimental systems

n/a	Involved in the study	
☒	☐	Antibodies
☒	☐	Eukaryotic cell lines
☒	☐	Palaeontology and archaeology
☒	☐	Animals and other organisms
☒	☐	Human research participants
☒	☐	Clinical data
☒	☐	Dual use research of concern

Methods

n/a	Involved in the study	
☒	☐	ChIP-seq
☒	☐	Flow cytometry
☒	☐	MRI-based neuroimaging
